# Glutamate excitotoxicity induced by orally administered propionic acid, a short chain fatty acid can be ameliorated by bee pollen

**DOI:** 10.1186/s12944-017-0485-7

**Published:** 2017-05-22

**Authors:** Afaf El-Ansary, Huda S. Al-Salem, Alqahtani Asma, Abeer Al-Dbass

**Affiliations:** 1 Central Laboratory, Female Center for Medical Studies and Scientific Section, King Saud University, Riyadh, Saudi Arabia; 2 Department of Pharmaceutical Chemistry, College of Pharmacy, King Saud University, Riyadh, Saudi Arabia; 3 Autism Research and Treatment Center, Riyadh, Saudi Arabia; 4 Shaik AL-Amodi Autism Research Chair, King Saud University, Riyadh, Saudi Arabia; 5 Department of Biochemistry, Science College, King Saud University, Riyadh, Saudi Arabia; 6 Medicinal Chemistry Department, National Research Centre, Dokki, Cairo, Egypt

**Keywords:** Propionic acid, Bee pollen, Glutamate excitotoxicity, Glutamine, GABA

## Abstract

**Background:**

Rodent models may guide investigations towards identifying either environmental neuro-toxicants or drugs with neuro-therapeutic effects. This work aims to study the therapeutic effects of bee pollen on brain glutamate excitotoxicity and the impaired glutamine-glutamate- gamma amino butyric acid (GABA) circuit induced by propionic acid (PPA), a short chain fatty acid, in rat pups.

**Methods:**

Twenty-four young male Western Albino rats 3–4 weeks of age, and 45–60 g body weight were enrolled in the present study. They were grouped into four equal groups: Group 1, the control received phosphate buffered saline at the same time of PPA adminstration; Group 2, received 750 mg/kg body weight divided into 3 equal daily doses and served as acute neurotoxic dose of PPA; Group 3, received 750 mg/kg body weight divided in 10 equal doses of 75 mg/kg body weight/day, and served as the sub-acute group; and Group 4, the therapeutic group, was treated with bee pollen (50 mg/kg body weight) for 30 days after acute PPA intoxication. GABA, glutamate and glutamine were measured in the brain homogenates of the four groups.

**Results:**

The results showed that PPA caused multiple signs of excitotoxicity, as measured by the elevation of glutamate and the glutamate/glutamine ratio and the decrease of GABA, glutamine and the GABA/glutamate ratio. Bee pollen was effective in counteracting the neurotoxic effects of PPA to a certain extent.

**Conclusion:**

In conclusion, bee pollen demonstrates ameliorating effects on glutamate excitotoxicity and the impaired glutamine-glutamate-GABA circuit as two etiological mechanisms in PPA-induced neurotoxicity.

## Background

Autism as a neurodevelopmental disorder is characterized by severe social impairment and communication, repetitive behavior, and restricted interests. Several studies recorded abnormality of glutamatergic signaling pathways in autism. Glutamate receptors are mainly localized in brain areas that have been repeatedly implicated in autism, such as the cerebellum and hippocampus [1] Excitatory glutamate signaling through glutamate receptors (GluRs) plays a key role in cortical development [2] which also indicate its involvement in the etiology of autistic features. Glutamate (Glu) is an amino acid that functions as an excitatory neurotransmitter in the central nervous system (CNS). Excitation causes depolarization of the postsynaptic neuron and promotes the propagation of action potentials. In contrast, GABA, the main inhibitory neurotransmitter, results in the hyperpolarization of the postsynaptic cell membrane, dampening the generation of action potentials.

Glutamate is usually synthesized in the neuron and is then transported into glial cells from the synaptic cleft, it is converted into Gln by the enzyme glutamine synthetase (GS), which is completely absent in neuronal cells [3]. Glutamine is then transported back into the neuron, where it is deaminated by glutaminase, once again forming Glu [3].

It is well known that Glu signaling is involved in a wide variety of psychological and cognitive processes. Alterations of this signaling have been associated with depression, learning disability and many neurodevelopmental disorders, including autism [4]. A balance between excitatory and inhibitory neurotransmission is critically important, and the loss of this balance is related to autism [5]. Several studies demonstrate the increased levels of plasma and brain Glu together with lower levels of GABA, Gln or their relative ratios to Glu (i.e., Glu/GABA and Glu/Gln) in autistic children, especially those with normal IQ [6]. Moreover, a relationship between Glu excitotoxicity and neuroinflammation in autism was ascertained and suggests that Glu signaling is an attractive target for the development of a therapeutic strategy for autism through Glu receptors and /or transporters, as important proteins playing a critical role in Glu homeostasis [7].

Propionic acid is a metabolite of clostridia species, bacteria found at higher levels in autistic feces compared to controls. Propionic acid was found to be capable of inducing biochemical, behavioral, electrophysiological, and neuropathological changes in rats similar to those observed in autistic patients when given either orally [8, 9] or through direct cerebroventricular infusion [10]. In our recently published work, serotonin, dopamine, and nor-adrenaline as three important neurotransmitters related to autism was remarkably impaired in PPA-treated juvenile rats and were also ameliorated with bee pollen [9]. Additionally, bee pollen demonstrated anti-inflammatory and anti-apoptotic effects through the reduction of PPA –induced levels of IFN-γ and caspase-3 as neuroinflammatory and apoptotic markers respectively [9].

Apitherapy is the medical use of honey bee products. This includes the use of honey, pollen, bee bread, propolis, royal jelly, and bee venom. Several studies on neuroinflammatory diseases in animal models have increasingly supported the effectiveness of this therapy in treating diseases related to microglia activation and Glu excitotoxicity among which is autism [11, 12]. The apitherapeutic mechanism of bee pollen was recently attributed to its antimicrobial activity and potency to induce the regeneration of damaged tissues [13]. It has also been shown that the ethyl alcohol extract of pollen recorded antibiotic activity against Gram-positive pathogenic bacteria, including *Klebsiella pneumonia (*A *propionibacterium)* and *Pseudomonas aeruginosa* and fungi such as *Candida albicans*. All these microbial organisms, together with Clostridia, are overgrown in most autistic patients, especially those frequently treated with ampicillin as a broad spectrum antibiotic. In addition, bee pollen is known to have detoxification activity, such as heavy metals (Mercury and lead), as a pathological mechanism involved in autism [14]. Pollen also acts through anti-inflammatory mechanisms through the inhibition of the activities of cyclooxygenase and lipoxygenase. These enzymes are responsible for the conversion of arachidonic acid into toxic compounds, such as prostaglandin and leukotrienes, which are inducers of acute and chronic inflammatory conditions and are biomarkers of autism [15].

It is well documented that Glu excitotoxicity is clinically related to neuroinflammation, oxidative stress, and apoptosis [16, 17]. In our recent study, bee pollen was found to be effective in ameliorating signs of PPA-induced brain toxicity, measured as a depletion of serotonin (5HT), dopamine and nor-adrenaline as important neurotransmitters related to autism, significant increase of IFN-γ and caspase 3 as markers of neuroinflammation and apoptosis respectively [18].

This information motivates our interest to investigate the therapeutic effects of bee pollen on Glu excitotoxicity-related parameters in rat pups orally administered PPA as a rodent model of autism [8]. Glutamate excitotoxicity was selected because it is ascertained as an etiological mechanism in autism directly related to the activation of microglia effectively treated with apitherapy [10, 19].

## Methods

**Fig. 1:**
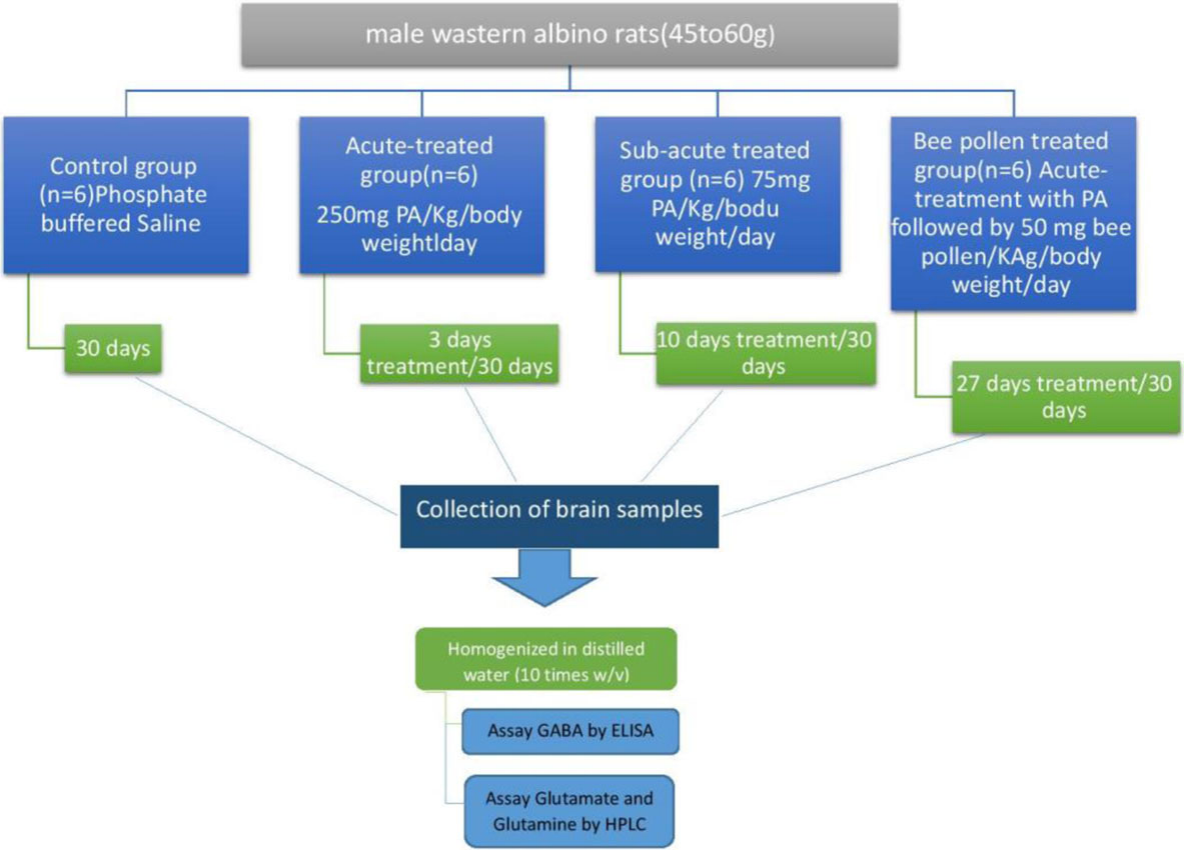
Schematic presentation of the experimental work demonstrating, dosage, duration of different studied groups

The experimental assays for this study were performed on 24 young (3–4 weeks of age) male western albino rats (45 to 60 g). Rats used in the present study were bred at the animal house of the pharmacy college, King Saud University, and were randomly assigned to four groups of six rats each. The first group consisted of rats to which only phosphate buffered saline was given at the time the PPA was administered to ensure that treatment administration is matched among comparison groups and was used as a control group. The second and third groups were orally administered a neurotoxic dose of propionic acid (PPA) (750 mg/kg body weight divided in 3 doses of 250 mg/kg body weight/day served as the acute group [8], and 750 mg/kg body weight divided in 10 equal doses of 75 mg/kg body weight/day served as the sub-acute group). The fourth group received an oral dose (50 mg/kg body weight for 30 days) of 100% natural bee pollen, imported from Wadi Al-Nahil, a marketing company in Saudi Arabia, after being treated with an acute dose of PPA. The four groups of rats were housed under controlled temperature (21 ± 1 °C) with ad libitum access to food and water. The animals were sacrificed at the end of each treatment. The protocol of the present work was approved by the Ethics Committee at the King Saud University and all experiments were performed in accordance with the guidelines of the National Animal Care and Use Committee. Figure 1, summarizes the experimental design of the present work.

### Tissue preparation

At the end of the experiment, the rats were anesthetized with carbon dioxide and decapitated. The brain was removed from the skull and was dissected into small pieces to be homogenized in distilled water (10 times *w*/*v*).

### Measurement of GABA levels

GABA was quantitatively determined using the ELISA immunoassay kit from ALPCO Diagnostics (Salem, NH, USA). Derivatization of extracted GABA was performed using the D-reagent. A quantity of 25 μL of the derivatives was then used for subsequent ELISA assays according to the provided instructions. This product has a wide detection limit range of 15–1500 ng/ml.

### Measurement of Glu and Gln levels

Glutamate and Gln levels were assessed using an HPLC method. Brain homogenate samples (0.1 ml) were mixed with 5 μl mercaptoethanol and allowed to stand for 5 min at room temperature, then precipitated with ice-cold methanol while being vortexed. Tubes were allowed to stand for 15 min in an ice bucket before the samples were separated by centrifugation (5000 rpm for 15 min) and the supernatant was collected. The protein-free supernatants were processed immediately for HPLC analysis of the two amino acids [20].

### Statistical analysis

The data were analyzed using the Statistical Package for the Social Sciences (SPSS, Chicago, IL, USA). The results were expressed as the means ± S.D. All statistical comparisons between the control and PA and pollen-treated rat groups were performed using the one-way analysis of variance (ANOVA) tests with Dunnett’s test for multiple comparisons. Significance was assigned at the level of *P* < 0.05. Receiver operating characteristics curve (ROC) analysis was performed. The area under the curve (AUC), cutoff values, and the degrees of specificity and sensitivity were calculated. Pearson’s correlations were performed between the measured parameters.

## Results

**Fig. 2:**
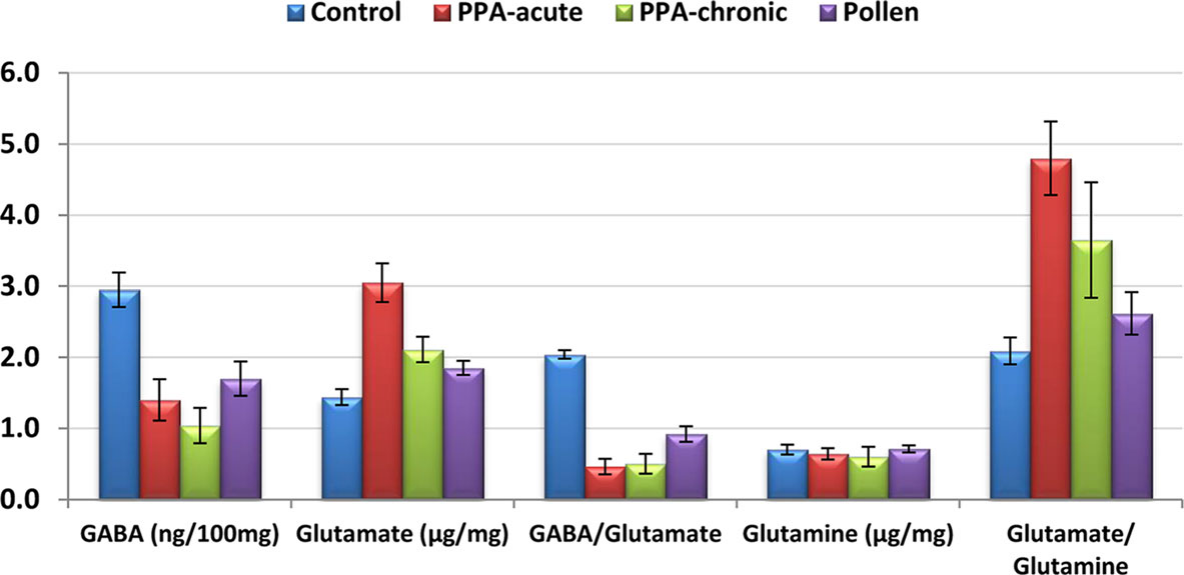
Mean ± S.D. and error bars of all parameters in all groups

**Fig. 3:**
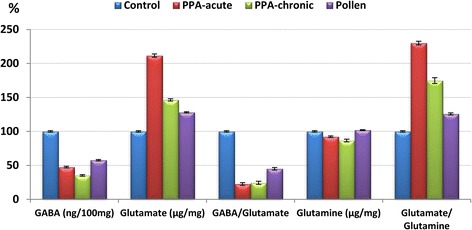
Percentage increase or decrease of the measured parameters relative to controls, diagrammatically presented as 100%

Results are presented as the means ± S.D. and the percentage change of at least six independent measurements. Table 1 and Fig. 2 present the mean ± S.D. of the absolute values of Glu, GABA, and Gln together with the two relative values of GABA/Glu and Glu/Gln in the brain homogenates of the four studied groups of rats. Compared to the control groups, the PPA-treated rats exhibited a significant increase in Glu and the Glu/Gln ratio with a concomitant decrease of GABA, Gln, and the GABA/Glu ratio. Figure 3 demonstrates the percentage change of the measured parameters relative to the control group. Glutamate showed 111.56% and 46.47% increases with acute and sub-acute PPA treatments, respectively. The decrease in GABA was more pronounced in sub-acute compared to acute samples, recording 64.74% and 52.46%, respectively. Glutamine was also much lower in sub-acute PPA-treated rat pups, recording a 13.42% decrease compared to a value of only 7.67% in acute-treated rats. The recorded decrease of GABA/Glu and increase of Glu/Gln ratios were more or less similar in both PPA-treated groups. The ameliorating effect of bee pollen was observed through the remarkable decrease of glutamate together with the increase of GABA and Gln in PPA-acute neuro-intoxicated rats.

**Table 1:** Mean ± S.D. of the absolute and relative concentrations of GABA, Glu, and Gln in control, PPA-intoxicated and PPA-intoxicated and pollen-treated rat pups

Parameter	Group	N	Mean ± S.D	Percent Change	*P* value^a^	*P* value^b^
GABA (ng/100 mg)	Control	6	2.95 ± 0.24	100.00		
	PPA-acute	6	1.40 ± 0.29	47.54	0.001	
	PPA-sub-acute	6	1.04 ± 0.25	35.26	0.001	0.042
	PPA –acute-Pollen	6	1.70 ± 0.24	57.67	0.001	0.079
Glu (μg/mg)	Control	6	1.44 ± 0.11	100.00		
	PPA-acute	6	3.05 ± 0.27	211.56	0.001	
	PPA-sub-acute	6	2.11 ± 0.18	146.47	0.001	0.001
	PPA –acute-Pollen	6	1.85 ± 0.10	128.09	0.05	0.001
GABA/Glu	Control	6	2.04 ± 0.06	100.00		
	PPA-acute	6	0.46 ± 0.11	22.67	0.001	
	PPA-sub-acute	6	0.50 ± 0.14	24.30	0.001	0.658
	PPA –acute-Pollen	6	0.92 ± 0.11	44.98	0.046	0.001
Gln (μg/mg)	Control	6	0.70 ± 0.07	100.00		
	PPA-acute	6	0.64 ± 0.08	92.33	0.272	
	PPA-sub-acute	6	0.60 ± 0.14	86.58	0.176	0.559
	PPA –acute-Pollen	6	0.71 ± 0.05	101.91	0.722	0.126
Glue/Gln	Control	6	2.09 ± 0.19	100.00		
	PPA-acute	6	4.80 ± 0.52	230.02	0.001	
	PPA- sub-acute	6	3.65 ± 0.81	174.83	0.004	0.015
	PPA –acute-Pollen	6	2.62 ± 0.30	125.78	0.004	0.001

^a^P value between the control group and other groups

^b^P value between the PPA-acute group and other groups

**Fig. 4:**
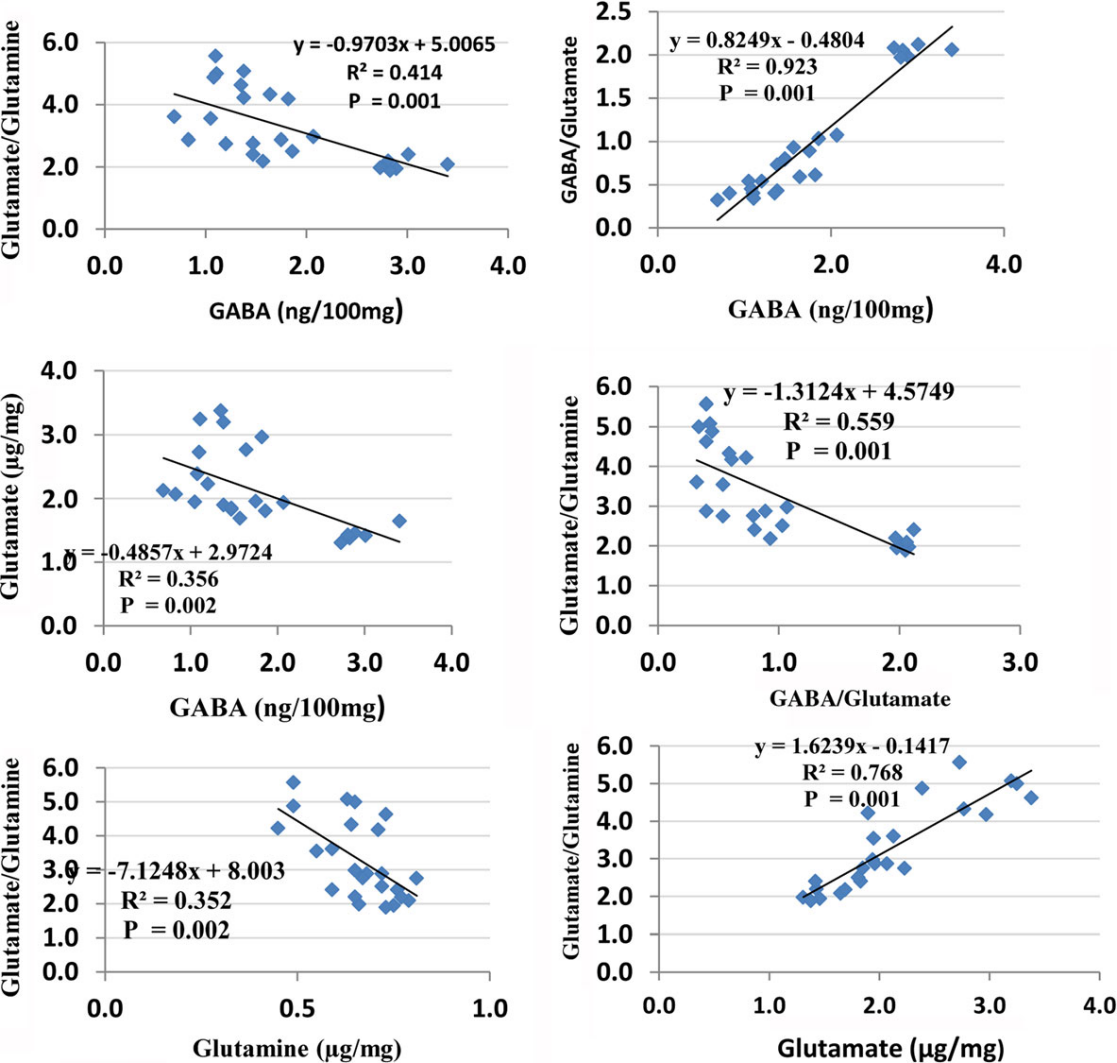
Positive and negative Pearson’s correlations between the measured parameters with a best fit line curve

Figure 4 shows the Pearson’s correlations between the three absolute values of the three measured parameters together with the relative concentrations of GABA/Glu and Glu/Gln. Negative and positive correlations are illustrated.

**Fig. 5:**
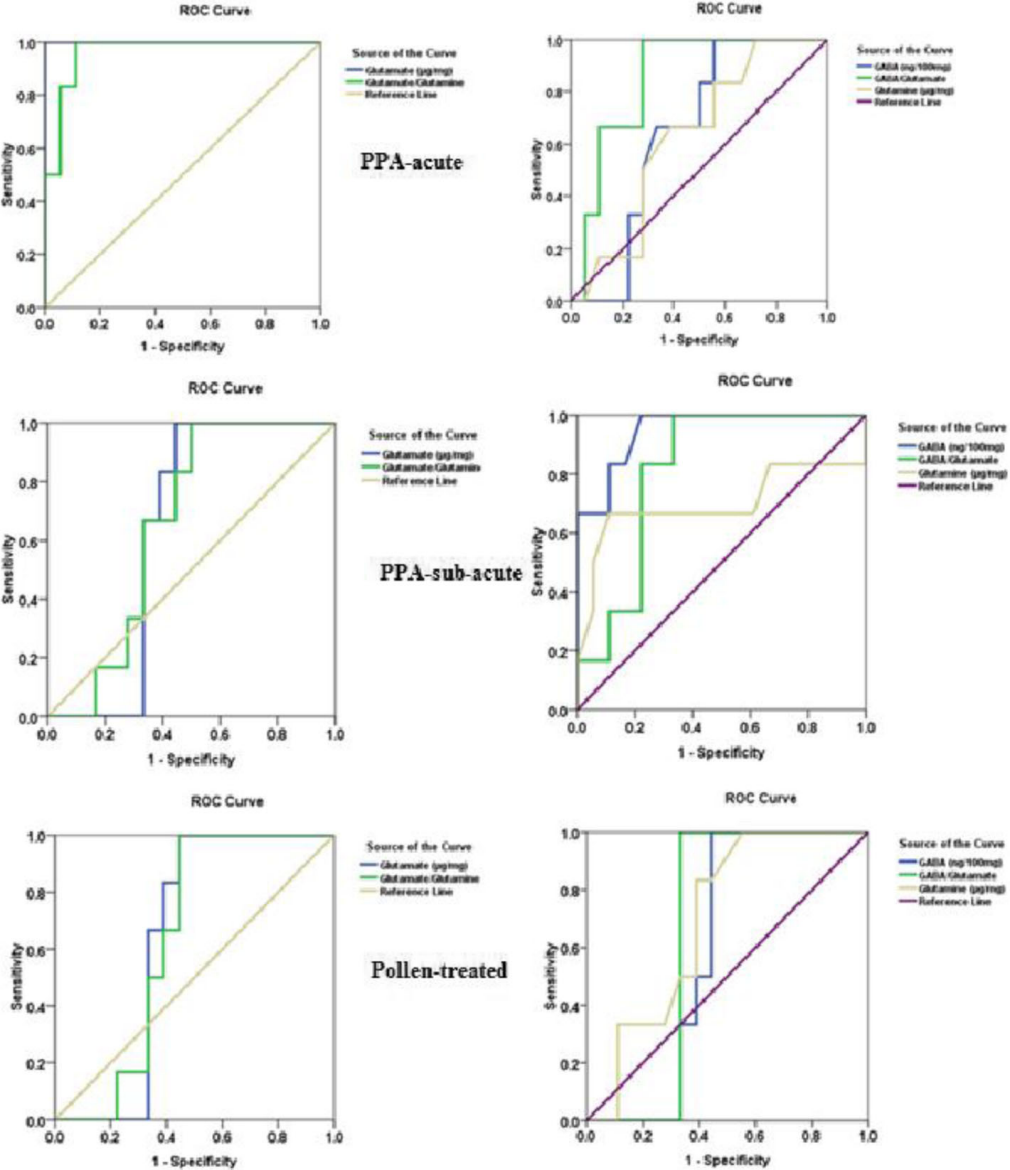
ROC curves of all parameters in PPA-chronic, PPA-acute or PPA-acute-followed with pollen as a treatment for glutamate neurotoxicity

Table 2 and Fig. 5 demonstrate the AUC, specificity, and sensitivity measured markers in PPA-acute and sub-acute intoxicated rats together with the pollen-treated group. It was shown that while glutamate and glutamine can be used as markers for PPA-acute neurotoxicity (AUC of almost 1) with 100% or near 100% sensitivity and specificity, GABA shows high validity as a marker for sub-acute toxicity (AUC = 0.949), with 100% sensitivity and 77.8% specificity. GABA/Glu showed equal predictiveness values for both modes of PPA intoxication with AUCs of 0.852 and 0.815 and satisfactory sensitivity and specificity. However, all the measured parameters demonstrate fair validity as biomarkers for pollen’s therapeutic effect (AUC range between 0.6–0.7).

**Table 2:** ROC curve of the measured absolute and relative parameters in all groups

Parameter	Group	Area under the curve	Best Cutoff value	Sensitivity %	Specificity %
GABA (ng/100 mg)	PPA-acute	0.653	1.840	100.0%	44.4%
	PPA-sub-acute	0.949	1.425	100.0%	77.8%
	Pollen	0.602	1.425	100.0%	55.6%
Glu (μg/mg)	PPA-acute	1.000	2.560	100.0%	100.0%
	PPA- sub-acute	0.639	1.875	100.0%	55.6%
	Pollen	0.639	2.015	100.0%	55.6%
GABA/Glu	PPA-acute	0.852	0.670	100.0%	72.2%
	PPA- sub-acute	0.815	0.761	100.0%	66.7%
	Pollen	0.667	0.761	100.0%	66.7%
Gln (μg/mg)	PPA-acute	0.630	0.655	66.7%	61.1%
	PPA- sub-acute	0.699	0.610	66.7%	88.9%
	Pollen	0.699	0.665	83.3%	61.1%
Glu/Gln	PPA-acute	0.963	3.897	100.0%	88.9%
	PPA- sub-acute	0.657	2.634	100.0%	50.0%
	Pollen	0.639	3.265	100.0%	55.6%

Table 3 demonstrates the multiple regression analysis using Gln as a dependent variable. It can be easily observed that the R^2^ value of 0.973 shows that 97.3% of the increase in Glu is related to an impaired Glu-Gln cycle through which Glu released from the neuron cells is transported to the astrocytes, where it can be converted to Gln.

**Table 3:** Multiple regression stepwise analysis with glutamate as dependent variable

Predictor Variable	Beta	*P* value	Adjusted R square	Model	
				F value	*P* value
Glutamate/Glutamine	1.211	0.001	0.973	410.254	0.001
Glutamine (μg/mg)	0.565	0.001			

## Discussion

It is well known that the increase of Glu can activate glutamate receptors, which may mediate excitotoxicity and lead to neuronal cell death. Microglial activation together with the overstimulation of Glu receptors has a principle role in Glu excitotoxicity, which might lead to abnormal brain development and synaptic plasticity and be implicated in PPA neurotoxicity [21].

The present study revealed that a PPA dose of 750 mg/kg body weight, given either acutely (250 mg/kg body wt. daily for three subsequent days) or sub-acutely given over a 10 days, can induce a significant increase of Glu in the rat brain. It was shown that while sub-acute PPA treatment induced a greater reduction of GABA and Gln as an inhibitory neurotransmitter and Glu buffering product, respectively, acute treatment was more effective in inducing a much higher increase in the excitatory Glu neurotransmitter.

In the present study, the involvement of Glu in PPA-induced neurotoxicity was examined because Glu is believed to play important roles in various types of neuronal death and its elevation might be due to leakage from dead cells. This can be supported by considering the previous work of El-Ansary et al. [8, 9] in which the same dose of PPA (750 mg/kg body wt.) was toxic enough to induce a significant increase of caspase 3 as a marker of brain cell death. Moreover, it can find more support in the recent histopathological study which proved that PPA- treatment induced severe cortical neuronal density with ballooning fibrillar acidophilic cytoplasm, degenerated, atrophied, and necrotic neurons together with amygdala mitochondrial crystalysis and microtubular distortion [22]. Both neuron necrosis and mitochondrial crystalysis as histopathological changes can be related to glutamate excitotoxicity. Upon exposure to glutamate, delayed calcium de-regulation usually followed by necrosis, and superoxide anions-induced mitochondrial dysfunction [23, 24].

GABA is derived from glutamate under the decarboxylation action of glutamate decarboxylase (GAD) via Gln-Glu-GABA circulation; thus, changes in the Glu level could affect the GABA content as well. Studies have found that a single dose of the severe neurotoxic compound amphetamine-type stimulant (ATS) (30 mg/kg) in mice down-regulated the Gln/Glu and GABA/Glu ratios, which suggested a circulatory disturbance of the Gln-Glu-GABA circuit [25]. In the present study, PPA demonstrated a level of neurotoxicity comparable to that of ATS [24]. The recorded changes in Glu-Gln -GABA circulation support the persistent autistic features induced in rat pups through the use of orally administered PPA [8, 9]. It was also related to the results of Tanaka et al. [26], who generated an animal model with behavioral and neuroanatomical abnormalities similar to those observed in autism through the over-stimulation of Glu receptors by the genetic down-regulation of the glial Glu transporters GLT1 and GLAST [26]. This may lead to a decreased uptake of glu from the synaptic cleft and result in excitotoxicity due to a significant elevation of the extracellular Glu level. Moreover, the significantly higher Glu/Gln and lower GABA/Glu ratios presented in Table 1 and Fig. 2 ascertain the effectiveness of PPA in inducing the rodent model of autism.

It is well known that the Glu-Gln cycle in the brain functions to control the levels of glutamate and to shuttle nitrogen between astrocytes and neurons. Under normal physiological conditions, Glu is metabolized in astrocytes by the GS reaction rather than by GAD, which favors Gln formation [26]. The remarkable increase of the Glu:Gln ratio in PPA-treated pups can be considered among the persistent autistic features related to PPA neurotoxicity and to the animal modeling of autism. This is supported by the work of Abu Shmais et al. [20], who recorded a significant increase in the Glu:Gln ratio in autistic patients compared to controls and suggested that the Glu-Gln cycle was greatly affected in these patients.

The significantly lower GABA level and GABA/Glu ratio found in PPA-treated pups could be caused either by a loss of GABAergic interneurons, a decrease in GABA synthesis, or alterations in the astrocytic cycling of GABA, Glu, and Gln [27]. All of these suggested mechanisms have been found as phenotypes in autism and are related to cognitive impairment [27, 28].

It is well accepted that oxidative stress and the extracellular release of Glu are reciprocally related [29]. Moreover, GS is highly sensitive to ROS-induced damage, which can also impair inhibitory processes, such as GABA-stimulated chloride uptake [29, 30]. Thus, the neurotoxic effect of PPA reported in the current study represented by the impaired Gln-Glu-GABA circuit may be related to its oxidative effect previously reported as the induction of lipid peroxidation, glutathione depletion, decreased catalase and increased glutathione-s-transferase, together with the impairment of energy metabolism [6, 7, 31].

Bee pollen has a wide variety of health protection functions due to its many active components, which are mainly composed of sugars, proteins, free amino acids, vitamins, flavonoids, and coenzyme Q10 [29]. As a natural product, pollen is rich with proteins (22.7%), essential fatty acids (5.1%), phospholipids (0.45%), phytosterols (1.5%), digestible carbohydrates (30.8%), reducing sugars (mainly fructose and glucose) (25.7%), phenolic compounds (1.6%), and flavonoids (3%). Pollen is a significant source of vitamins (1.4%), such as vitamins E, A, D, B complex, folic acid and biotin [32, 33]. Most of these components were individually effective either in improving behavior in valproic acid rat model, or clinically in reducing symptoms of neurodevelopmental disorders among which is autism [33–37].

Table 1 and Figs. 2 and 3 demonstrate the ameliorating effect of bee pollen on the Gln-Glu-GABA circuit as a neurotoxic effect of PPA. It was observed that the therapeutic effect of bee pollen is presented as a less significant difference in the measured parameters compared to controls than that recorded by both groups of PPA-treated rat pups. Based on the reciprocal relationship between Glu excitotoxicity and oxidative stress, the therapeutic effect of bee pollen can be attributed to its antioxidant effect. This can be supported by considering the antioxidant and neuroprotective effects of coenzyme Q10 as a component of bee pollen [30]. This suggestion is in good agreement with the recent work of Al-Ghamdi et al. [38], which demonstrated that coenzyme Q10 has potential protective and restorative effects against PPA-induced brain injury, as confirmed by improvements in biochemical markers and DNA double-strand breaks.

Moreover, the reported therapeutic effect of bee pollen can also be attributed to its content of flavonoids as one of the most potent antioxidant compounds. The amelioration of glutamate toxicity measured as (Glu / Gln) ratio, is consistent with the study of Yang et al. [39] and Kim et al. [40] which ascertain the role of flavonoids as major component of bee pollen in the attenuation of glutamate-induced neurotoxicity. This can find more support through considering the fact that flavonoids and their metabolites have been found in the brain tissues of rodents after oral administration [41], suggesting their ability to cross the blood–brain barrier.

The positive and negative correlations between the measured parameters presented in Fig. 4 ascertain the importance of the Glu-Gln-GABA circuit as a persistent biochemical autistic feature, which can be targeted to ameliorate the neurotoxic effect of PPA.

In receiver operating characteristics (ROC) analysis, while an AUC value close to 1 was an excellent diagnostic and predictive marker, a curve that lay close to the diagonal (AUC = 0.5) had no diagnostic utility. An AUC value close to 1 was always accompanied by satisfactory values of specificity and sensitivity of the biomarker. Table 2 and Fig. 5 demonstrate that among the three measured parameters, while GABA can be used as perfect marker for sub-acute PPA neurotoxicity (AUC = 0.949), was recorded (AUC = 1) in cases of acute treatment with PPA. The GABA/Glu ratio recorded more or less the same predictive value for both modes of toxicity. Glutamine was the least predictive recorded AUC value in the range of 0.6–0.7, which showed a relative predictive value for the three treated groups (acute, sub-acute, and pollen-treated). Table 3 also shows that GABA, Glu, GABA/Glu, Gln, and Glu/Gln can be used as fair markers for the ameliorating effect of bee pollen treatment (AUCs 0.6–0.7).

## Conclusion

These findings suggest that bee pollen is an attractive candidate for further studies for the development of new treatment strategies that can target glutamate excitotoxicity and oxidative stress as two etiological mechanisms of autism. Higher doses of bee pollen may yield more ameliorating effects, but because pollen is heavily under-researched it cannot be recommended for any particular usage in autistic patients until more studies are conducted.

## Abbreviations

AUC: Area under the curve
CNS: Central Nervous System
GABA: Gamma aminobutyric acid
Gln: Glutamine
Glu: Glutamate
GS: Glutamine synthetase
PPA: Propionic acid
ROC: Receiver Operating Characteristics
